# Describing and Simulating Concurrent Quantum Systems

**DOI:** 10.1007/978-3-030-45237-7_16

**Published:** 2020-03-13

**Authors:** Richard Bornat, Jaap Boender, Florian Kammueller, Guillaume Poly, Rajagopal Nagarajan

**Affiliations:** 8 Johannes Kepler University, Linz, Austria; 9 University of Birmingham, Birmingham, UK; 10 Department of Computer Science, Middlesex University, London, UK; 11 Hensoldt Cyber GmbH, Taufkirchen, Germany; 12 Widmee, Région de Bordeaux, Bordeaux, France

## Abstract

We present a programming language for describing and analysing concurrent quantum systems. We have an interpreter for programs in the language, using a symbolic rather than a numeric calculator, and we give its performance on examples from quantum communication and cryptography.

Quantum cryptographic protocols such as BB84 QKD [3] and E92 QKD [7] offer unconditional statistical security. These protocols have been implemented in commercial products; various QKD networks have been built around the world; and China has launched a dedicated satellite for quantum communication. The security of the protocols has been established information-theoretically, but their implementations may have security loopholes. We intend to investigate the security question, eventually by using formal methods to verify the properties of implementations, but first by simulation of protocols expressed as programs.

Large companies are developing full-stack solutions for implementing quantum algorithms, and quantum computers will likely be network-linked. Although we have focused on quantum communication and cryptography protocols, aspects of our work will be applicable to distributed quantum computation.

Concurrent quantum systems, such as communication and cryptographic protocols assume physically-separated *agents* (Alice, Bob, etc.) who communicate by sending each other *qubits* (quantum bits: polarised photons, for example) and classical bit-strings. There are a few dedicated, high-level programming languages for quantum systems such as Microsoft's Q# [2]. They focus on single-machine computation and lack a treatment of communication, but a protocol simulation must ensure, for example, that a qubit transferred from one agent to another can't be used again by the sender and can't be used by the receiver before it is sent. We decided therefore to take a process-calculus approach, and we have implemented a tool inspired by CQP [9]. Our implementation is called qtpi [1], and uses symbolic rather than numeric quantum calculation. Programs are checked statically, before they run, to ensure that they obey real-world restrictions on the use of qubits (no cloning, no sharing). Unlike CQP, which preserves all possible outcomes, labelling each with a probability, qtpi takes a single execution path, making probabilistic choices between outcomes.

We have used qtpi to simulate simple protocols such as teleportation, and some more involved ones including the quantum key-distribution protocols BB84 [3] and E92 [7]. Each of these involves transmission of qubits and public transmission of classical messages (in the case of BB84, over an authenticated channel [13]), all of which is simulated. It is early days in our development of the tool, so there is as yet no provision for formal proof, but in these examples we can already simulate well over 1M qubit transfers per minute on a small laptop – i.e. we can simulate largish examples in a useful time.

## 1 Processes

Protocols are carried out by *agents* which send each other messages but share no other information. We simulate agents by *processes* which share no data or variables. Typical protocol steps from the literature are

– obtain a qubit, perhaps initialised to one of $|0\rangle$, $|1\rangle$, $|+\rangle$ or $|-\rangle$;
– put a qubit through a gate such as I, H, X, etc.;
– measure a qubit;
– send or receive a qubit;
– send or receive a classical value, such as a list of numbers or bits.

In addition an agent may perform a calculation, such as generating 1000 random bits or encrypting/decrypting a message or checking the values received in a message. Calculations aren't protocol steps and don't affect qubit state, though they often depend on the results of measuring qubits and their results often influence subsequent protocol steps. Our processes have analogues of protocol steps and calculations. In addition we are able to create processes, to choose conditionally between different processes and to set up a collection of processes running simultaneously.

The aim of our work is to mathematically analyse programs which describe quantum systems. Towards that end we have a semantics of quantum-mechanical calculation [5], written in Coq [10]. That is work in progress: for the time being we are able to execute our protocol-programs using our simulator [1].

### 1.1 A programming language

Our language has two distinct notations: a protocol-step language, which is derived from the pi-calculus [11], and a functional calculation language, somewhat in the style of Miranda [12]. Neither language has assignment, although qubit measurement does change program state and so needs special attention. The protocol-step language has recursion, but only tail recursion: i.e. nothing can follow a process invocation step (but note that parallel execution of sub-processes provides more complexity).

Following the pi-calculus we use *channels* to communicate between processes. So Alice doesn't send to Bob, she sends down a channel which Bob can read from – or perhaps it might be Eve, if there is interference. Channels are values, so you can set up communication between two processes by giving them the same channel-argument when you create them, and you can send channel values in messages to alter connections dynamically.

In the protocol-step language steps are separated by dots ('.') and choices are made between processes rather than single or multiple steps. Channels are created by (new $c$); send is $C!E, .., E$; receive is $C?(x, .., x)$; qubits are created by (newq $q$); quantum gating is $Q, .., Q \gg G$; quantum measurement $Q{-}/{-}(x)$.

In the expression language there is function application ($f$ $arg$), arithmetic and Boolean calculation, conditional choice and recursion. It uses infinite-precision rationals for numerical calculations.

## 1.2 Symbolic quantum calculation

Quantum calculations can be described using *quantum circuits*: diagrams such as Fig. 1 show how qubits (one per line) are put through gates (boxes, line-connectors) and/or measured (meter symbols) giving a classical 0/1 result.

In quantum mechanics the state of a qubit is a vector $a\,|0\rangle + b\,|1\rangle$, with $|a|^2 + |b|^2 = 1$. Here $|0\rangle$ and $|1\rangle$ are the computational basis vectors, $a$ and $b$ are complex amplitudes, and $|a|^2$ and $|b|^2$ give the probability of measuring the state as $|0\rangle$ or $|1\rangle$. In qtpi a single isolated qubit is therefore a pair of complex numbers, and quantum gates, such as the H, X and Z gates of Fig. 1, are square matrices of complex numbers which modify the state by multiplication. The state of $n$ entangled qubits is a $2^n$-element vector, matrices which manipulate all of it have to be $2^n \times 2^n$, so calculations with large entanglements can rapidly grow out of the range of straightforward simulation. Luckily, quantum security protocols typically work with a small number of qubits at a time.

Because our calculations are simple, we can afford to implement them symbolically. We use $h$ for $\sqrt{1/2}$; it is also equal to $\sin(\pi/4)$ and $\cos(\pi/4)$. A great deal of formulae can be expressed in terms of powers of $h$: for example $\cos(\pi/8)$ $= \sqrt{(1+h)/2}$.

Symbolic calculation involves lots of symbolic simplification. That makes it relatively slow, compared to calculation with floating-point numbers, but it is absolutely accurate – $h^2 + h^2$, for example, is exactly 1. When measuring, we must convert symbolic probabilities into numbers. But that is part of a statistical calculation, so minor inaccuracy is acceptable.

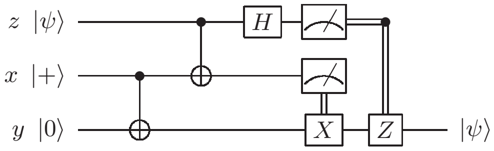

**Fig. 1.** Quantum circuit for teleportation

```
proc System () =
  (newq x=|+>, y=|0>) x,y>>CNot .
  (new c:^bit*bit) | Alice(x,c) | Bob(y,c)

proc Alice (x:qbit, c:^bit*bit) =
    (newq z)
    out!["initially Alice's z is "] . outq!(qval z) . out!["\n"] .
    z,x>>CNot . z>>H . z-/-(vz) . x-/-(vx) . c!vz,vx . _0

proc Bob(y:qbit, c:^bit*bit) =
    c?(b1,b2) .
    y >> match b1,b2 . + 0b0,0b0 . I
                       + 0b0,0b1 . X
                       + 0b1,0b0 . Z
                       + 0b1,0b1 . Z*X .
    out!["finally Bob's y is "] . outq!(qval y) . out!["\n"] . _0
```

**Fig. 2.** Teleportation of an unknown quantum state, with logging

### 1.3 No cloning

In the real quantum world there is no way of cloning a qubit – you can't start with a qubit in some arbitrary state and finish up with two qubits in that state. That, plus the fact that measurement irrevocably alters a qubit's state, is what provides quantum security protocols with unconditional security – though the uncertainty of measurement means that the guarantee is probabilistic, not absolute. A programming language which simulates quantum effects should therefore not allow copying of the value of a qubit variable. We use language restrictions to facilitate anti-cloning checks: in particular we severely restrict the use of qubits in data structures, in messages, and after measurement or transmission. Those checks are partly implemented by typechecking, partly by an efficient static symbolic execution before simulation begins.

### 1.4 Other notable features

Randomised priority queues of runnable processes and waiting communication offers ensure non-deterministic execution, and are used to eliminate infinite unfairness. Logging steps can be pushed into subprocesses to clarify protocol descriptions, leaving a marker in the logged process to show where it should occur (see examples in artifact [6]). Type descriptions are almost entirely optional.

## 2 Straightforward description

Our aim is to provide a programming language in which protocol descriptions are transparently easy to read. For example, Fig. 2 shows teleportation [4] using three processes: Alice and Bob carry out the protocol, and System sets up the communication between them. The calculation follows the circuit in Fig. 1, but is shared between agents obeying the anti-cloning restrictions.

The System process creates qubits $x$ and $y$ (`newq` ..), initialised to $|+\rangle$ and $|0\rangle$, and entangles them using a CNot gate (`x,y>>` ..). It creates a channel $c$ which carries pairs of bits (`new c` ..), and then splits into two subprocesses: one becomes Alice, taking one of the qubits and the channel; the other becomes Bob, with the other qubit and the same channel. Those processes run in parallel.

The Alice process creates a new qubit $z$, *without specifying its state*, and logs that state (the anti-cloning restrictions make this tricky). Then it puts $z$ and $x$ through a CNot gate (`z,x>>` ..), puts $z$ alone through a Hadamard gate (`z>>H`), and finally measures first $z$ (`z-/-`(vz)), then $x$ (`x-/-`(vx)), giving bits $vz$ and $vx$. Finally it sends those bits to Bob on the $c$ channel (`c!`...). The overall effect is subtle, because first System's actions entangle $x$ and $y$, so that measurement of $x$ constrains $y$, and then Alice entangles $z$, $x$ and $y$, so that measurement of $z$ constrains both $x$ and $y$.

The Bob process waits to receive Alice's message (`c?` ..), and calculates a gate (`match` ..) to process the results depending on one of four possibilities for the two bits it receives (note one of the gates is the matrix product of $Z$ and $X$). It puts $y$ through that gate (`y>>` ..) and logs the result. The output of this program is always

```
initially Alice's z is 2:(a2|0>+b2|1>)
finally Bob's y is 1:(a2|0>+b2|1>)
```

where $a_2$ and $b_2$ are unknown symbolic amplitudes. A sample execution trace, edited for brevity, shows the states produced by Alice's actions: qubit 0 is $x$, 1 is $y$, 2 is $z$; initially 0 and 1 are entangled, and the first step entangles all three.

```
Alice (2:(a2|0>+b2|1>),0:[0;1](h|00>+h|11>)) >> Cnot;
  result (2:[2;0;1](h*a2|000>+h*a2|011>+h*b2|101>+h*b2|110>),
          0:[2;0;1](h*a2|000>+h*a2|011>+h*b2|101>+h*b2|110>))
Alice 2:[2;0;1](h*a2|000>+h*a2|011>+h*b2|101>+h*b2|110>) >> H;
  result 2:[2;0;1]
            (h(2)*a2|000>+h(2)*b2|001>+h(2)*b2|010>+h(2)*a2|011>
             +h(2)*a2|100>-h(2)*b2|101>-h(2)*b2|110>+h(2)*a2|111>)
Alice: 2: (.. as above ..) -/- ;
  result 0 and (0:[0;1](h*a2|00>+h*b2|01>+h*b2|10>+h*a2|11>),
                1:[0;1](h*a2|00>+h*b2|01>+h*b2|10>+h*a2|11>))
Alice: 0:[0;1](h*a2|00>+h*b2|01>+h*b2|10>+h*a2|11>) -/- ;
  result 1 and 1:(b2|0>+a2|1>)
Chan 2: Alice -> Bob (0,1)
Bob 1:(b2|0>+a2|1>) >> X; result 1:(a2|0>+b2|1>)
```

Tracing several executions shows that Alice's measurements don't always give the same results in $vz$, $vx$ and qubit 1, so Bob doesn't always use the same gate(s). The qubit $z$ is never sent in a message, is destroyed by Alice's measurement, and its amplitudes are unknown to the program, but $y$ always finishes up in the state that $z$ began in. Without symbolic calculation we couldn't do such a simulation.

## 3 Performance on examples

We can run various simulations of the quantum key-distribution protocol BB84 [3], with Alice and Bob and various Eve processes. In order to generate a one-time key to encrypt an $n$-bit message, Alice needs to send many more bits than $n$, and our simulation allows us to experiment with various parameters of her calculation to see what happens. Here is a shortened display of part of the output of an example simulation (timing measurements made on VirtualBox Ubuntu 18.10, on a 7-year-old MacBook Air with 8GB RAM):

```
length of message? 4000; length of a hash key? 40;
minimum number of checkbits? 500; number of sigmas? 10;
number of trials? 100

13718 qubits per trial; 0 interfered with; 100 succeeded
```

It takes about 0.6 seconds for each trial, but overall it makes 1.3M qubit transfers and measurements in 60 CPU seconds. With an intercept-and-resend Eve, the same exchanges take 95 seconds, but Eve's interference is detected every time. With a very short message and very few checkbits we can show that even such a naive Eve can sometimes win, as statistical analysis predicts.

Our simulation of E92 QKD [7] uses 20 000 entangled qubit pairs per trial for the same-sized problem. Because the protocol calculations are more complicated and our calculation language is interpreted rather than compiled, simulation takes over 4 CPU minutes.

Qtpi can handle larger entanglements. In about 13 seconds it's able to set up and measure one 'brick' (ten qubits, all CZ-entangled) of the measurement-based quantum computing mechanism in [8] – but that's too small to be useful, and larger entanglements are exponentially worse.

## 4 Conclusions

We have a quantum programming language which allows description of protocols with multiple agents. It has protection, built from well-understood computer science foundations, against cloning of qubits within a simulation. It is not yet able to deal efficiently with entanglements of more than a few qubits. Its symbolic calculator is fast enough for the protocols we have examined.

## 5 Data Availability and Acknowledgements

The qtpi interpreter and the examples referred to in the paper are available at https://doi.org/10.6084/m9.figshare.11882592. Our research was supported by UK National Cyber Security Centre through the VeTSS project "Formal Verification of Quantum Security Protocols using Coq". Nagarajan was also supported by EU Cost Action IC1405 "Reversible Computation - Extending Horizons of Computing". We thank Simon Gay for helpful discussions.
